# Robotic-Assisted Laparoscopic Autotransplantation of the Left Kidney for Extended Iatrogenic Ureteral Injury

**DOI:** 10.7759/cureus.115288

**Published:** 2026-08-27

**Authors:** Franca Bergfelder, Fabian Rössler, Jose Oberholzer

**Affiliations:** 1 Surgery, Chirurgie Zentrum St. Anna, Luzern, CHE; 2 Surgery and Transplantation, Universitätsspital Zürich, Zürich, CHE

**Keywords:** autologous kidney transplantation, autotransplantation of the kidney, iatrogenic ureteral injury, nephrostomy, ureteral reconstruction

## Abstract

Iatrogenic ureteral injury is a rare but serious complication of lower abdominal surgery. In cases of extensive damage, conventional reconstruction may be unfeasible. Autologous kidney transplantation can preserve renal function in selected patients. A 57-year-old patient developed a long-segment left ureteral injury following laparoscopic hysterectomy with bilateral adnexectomy. A nephrostomy had been in place for over one year and was complicated by recurrent infections with progressive deterioration of kidney function. Due to the extent of the injury, direct reconstruction was not possible. Autologous kidney transplantation to the left iliac fossa with ureterocystostomy was performed using a robotic-assisted laparoscopic approach. Both nephrectomy and autotransplantation were completed without intra- or postoperative complications, and long-term kidney function remained stable. Autologous kidney transplantation is an effective option for complex ureteral injuries when conventional reconstruction is not feasible. Robotic assistance allows a safe minimally invasive approach with favorable functional outcomes.

## Introduction

Ureter injuries during extensive lymphadenectomy in gynecological surgery are uncommon but can result in severe complications. These injuries carry a high risk of recurrent urinary tract infections and can lead to a dramatic deterioration in kidney function. Minor or short injuries can often be managed without surgery through double-J catheters and nephrostomy placement alone [1]. However, long-segment ureteral defects pose a significant challenge. In such cases, long-term nephrostomy may be necessary. However, they are associated with an impaired quality of life and an increased risk of recurrent infections, with subsequent impairment of kidney function [2,3]. Techniques for ureter reconstructions depend on the length of ureter injury and may comprise bladder lift with direct anastomosis, or placement of a jejunal interposition graft [4]. However, these techniques may be ineffective if the remaining ureteral stump is too short, or if recurrent infections occur, as was the case with our patient. In such complex rare cases, autotransplantation of the left kidney, to ensure direct anastomosis of the short ureteral stump to the bladder, offers a potential curative solution.

## Case presentation

A 57-year-old woman was referred to our department by the urologists. One year prior, the patient had undergone a laparoscopic hysterectomy with bilateral adnexectomy and extended lymphadenectomy for ovarian cancer. During this procedure, a long-distance injury to the left ureter occurred, which was only noticed after the operation and required nephrostomy insertion. However, she suffered from recurrent infections, necessitating multiple changes of the nephrostomy and long-term antibiotic treatment. As a result, kidney function deteriorated from normal to a creatinine-based estimated glomerular filtration rate (eGFR) of 65 mL/min. Urologists defined a direct ureteral reconstruction or bladder lift as not feasible due to the extent of the ureteral defect and scarred conditions after lymphadenectomy. A jejunal interposition graft (uretero-ileo-ureterostomy) was considered too dangerous on account of the repeated infections. The nephrostomy, however, caused recurrent pyelonephritis, significant patient discomfort, and reduced quality of life. Magnetic resonance imaging (MRI) confirmed good perfusion and preserved function of the left kidney. A complementary kidney scintigraphy showed equal function of both kidneys. Following interdisciplinary discussion, we opted for minimally invasive kidney autotransplantation.

Surgical technique

**Figure 1:**
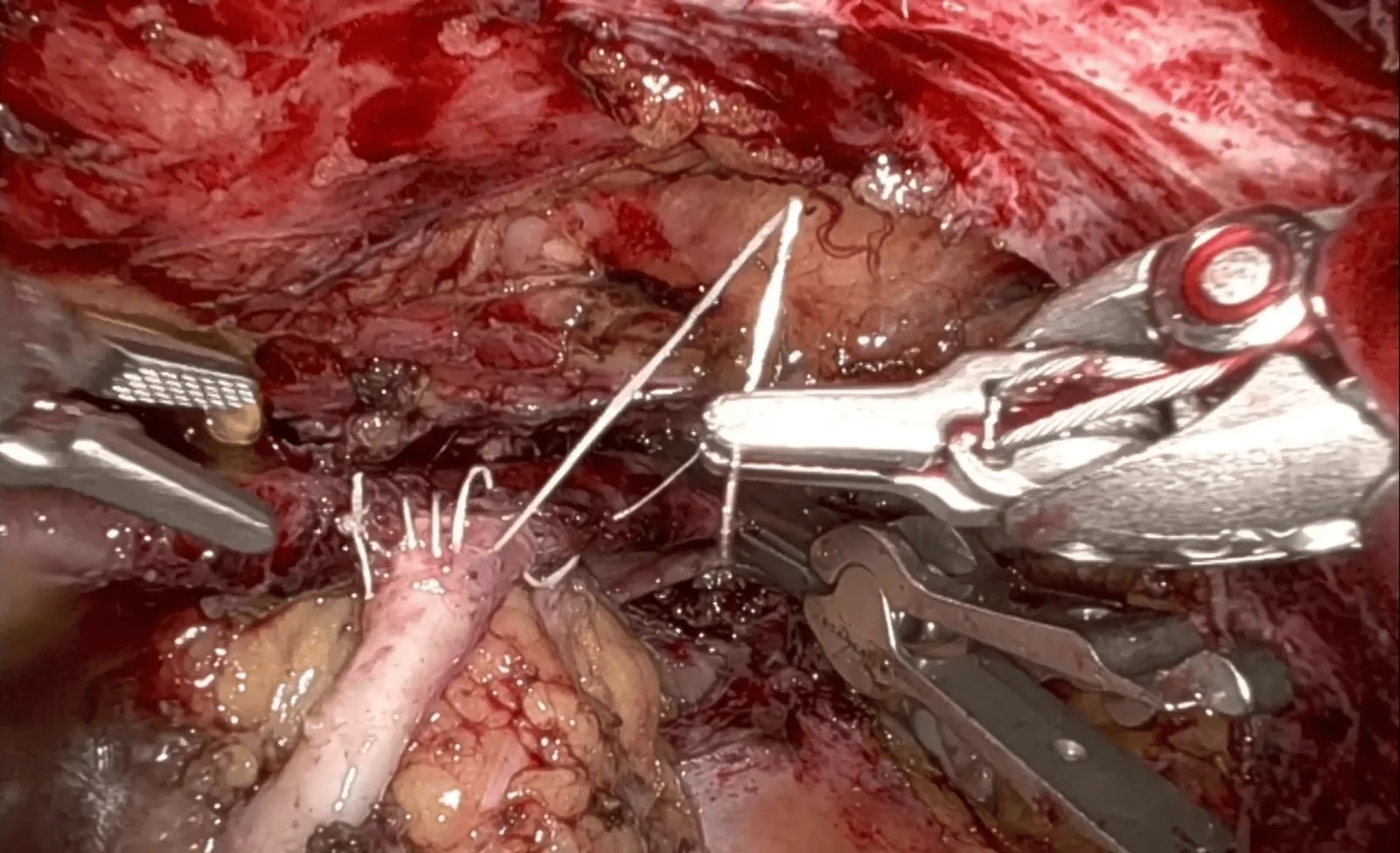
Intraoperative view of the arterial anastomosis during kidney transplantation The end-to-side anastomosis of the graft renal artery is performed using a running suture technique.

Both surgeries, left nephrectomy and autotransplantation to the left iliac axis, were performed laparoscopically, with a robotic-assisted technique using the Da Vinci Xi® Surgical System (Intuitive Surgical, Inc., Sunnyvale, CA, USA). Via a 6-cm infraumbilical minilaparotomy, the GelPort® (Applied Medical Resources Corporation, Rancho Santa Margarita, CA, USA) was inserted, through which an AirSeal® trocar (CONMED Corporation, Largo, FL, USA) was introduced. In addition, two 8-mm trocars were placed in the left epigastrium and in the left lateral subcostal area. A third 8-mm trocar was inserted through the GelPort®. Dissection was performed using the bipolar forceps on the left, and the monopolar hook on the right. The dissection of the left kidney was complicated due to adhesions caused by the chronic urinoma and recurrent infections and previous placement of a mesh for incisional hernias. The proximal ureter stump was located and preserved via antegrade filling with methylene blue through the nephrostomy. The renal artery and renal vein were dissected to the level of the aorta and transected using a vascular stapler (Echelon Flex^TM^ vascular stapler, Ethicon, Inc., Cincinnati, OH, USA). After extraction, the kidney was immediately flushed with cold perfusion solution (Institute George Lopez - IGL-2®, Lissieu, France) to remove the remaining blood. Warm ischemia time was three minutes. Subsequently, the kidney was preserved with a static cold storage technique. For subsequent autotransplantation, no extra trocars were needed. Vascular dissection of the left iliac axis was challenging due to previous lymphadenectomy, but the actual vessel quality was good. Autotransplantation to the left iliac fossa was performed with arterial (Figure 1) and venous end-to-side anastomoses to the external iliac vessels using Goretex sutures.

**Figure 2:**
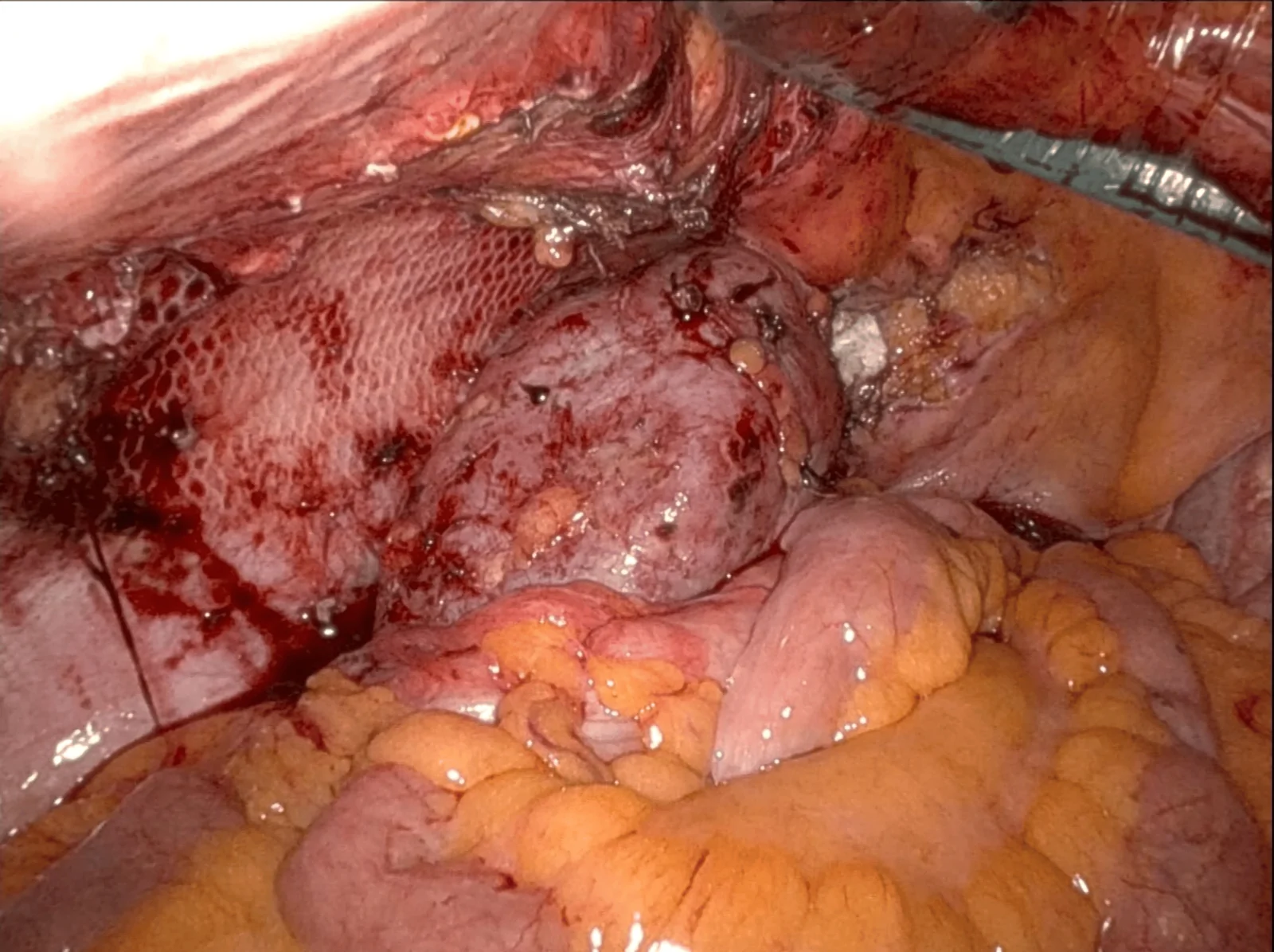
Situs following renal graft implantation into the left iliac fossa, with completed arterial anastomosis

Reperfusion was uneventful, with an anastomosis time of 41 minutes and cold ischemia time of 2 hours and 44 minutes (Figure 2).

**Figure 3:**
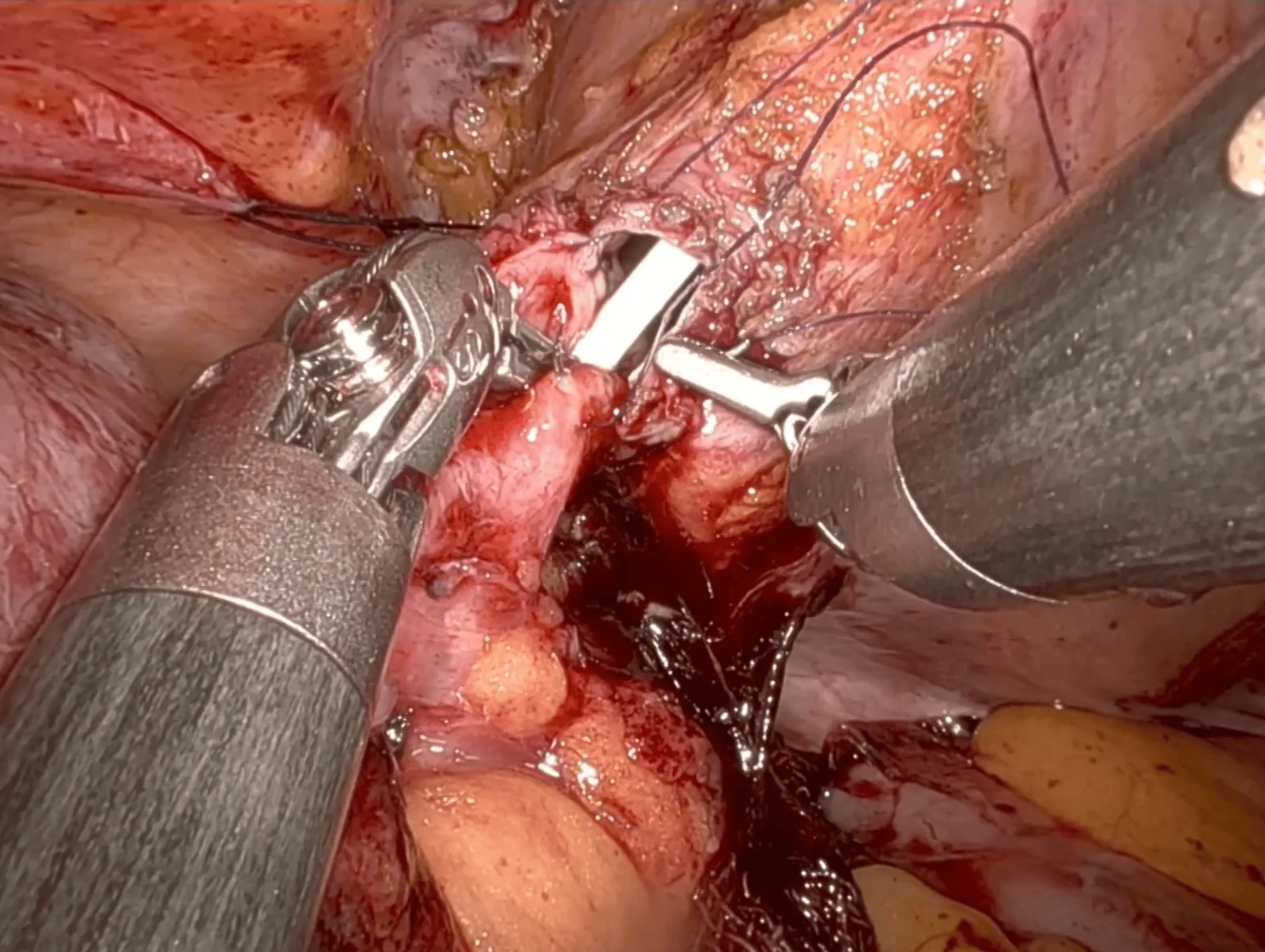
Uretero-vesical anastomosis using the Lich-Grégoir extravesical technique The ureter is spatulated and anastomosed to the bladder mucosa through a submucosal tunnel, secured with interrupted sutures to create an antireflux mechanism, without opening the bladder lumen.

The bladder was mobilized laterally to allow for direct anastomosis with the short ureter stump. Ureterocystostomy was performed using the Lich-Grégoir technique, with a reflux-plasty and placement of a double-J catheter (Figure 3).

Postoperative course

**Figure 4:**
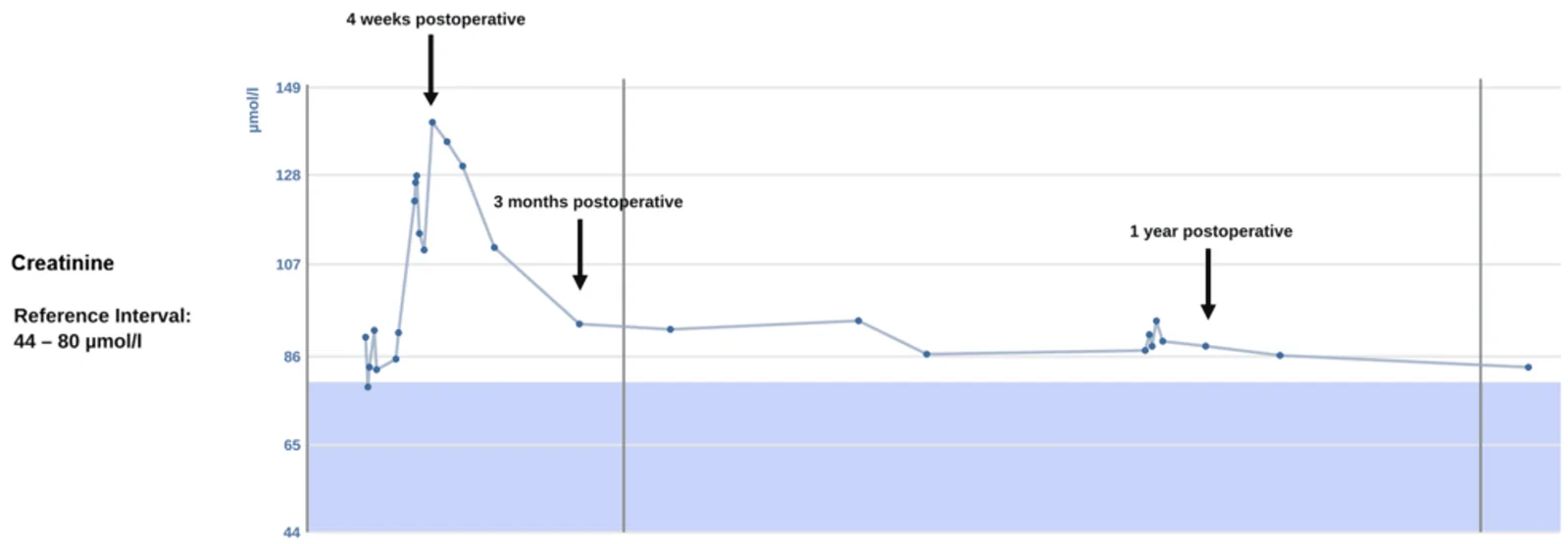
Serum creatinine trend after kidney transplantation, showing a transient rise at four weeks postoperatively, followed by normalization and a stable course through one year

**Figure 5:**
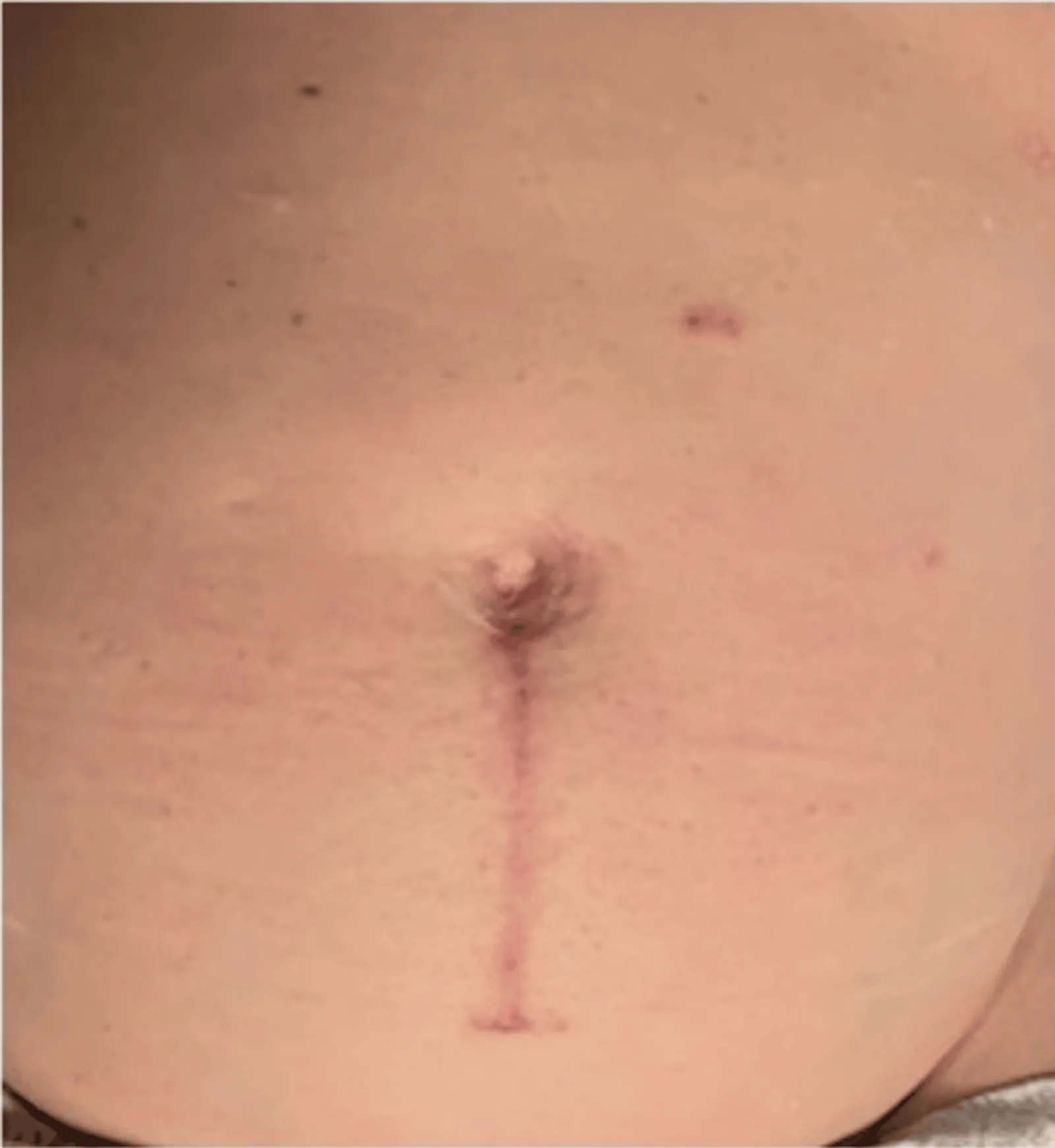
Umbilical trocar scar at six months following da Vinci-assisted kidney transplantation, showing good cosmetic outcome

The intra- and early postoperative course was uneventful, and the patient recovered in a timely manner. Postoperative sonography confirmed regular perfusion and no signs of ureteral obstruction. The patient was discharged on postoperative day 7 in good general condition and improved kidney function. Three weeks postoperatively, the patient presented with significant weight gain, dyspnea, and deterioration of kidney function. CT imaging revealed four-quadrant ascites, suspicious for lymph fistula. Kidney scintigraphy showed no evidence of urinoma or leakage. With dietary measures (fat-reduced diet with MCT supplementation) and diuretic therapy, symptoms quickly regressed. We did not place any percutaneous drainage to avoid transcutaneous fistula formation. Kidney function improved gradually (Figure 4). No urinary tract infections occurred in the postoperative course. Figure 5 shows the postoperative cosmetic outcome six months after surgery.

## Discussion

We present a rare case of robotic-assisted laparoscopic left kidney autotransplantation, as treatment for a large, complex iatrogenic ureteral injury. Surgery was uneventful and successful, and with the use of robotic-assisted surgery, we could perform both procedures, kidney explantation and reimplantation, via the same small surgical access. This case highlights the importance of performing such procedures in high-volume transplant centers with specialization in complex transplantations and urological reconstructions. With 120 kidney transplantations performed annually and frequent use of robotic-assisted surgery for living donor nephrectomies and laparoscopic kidney transplantations, our center has extensive experience with minimally invasive kidney transplant surgery.

Long-segment ureteral injuries remain challenging, particularly when a conventional ureteral reconstruction (e.g., uretero-ureterostomy or ileal interposition) is not feasible. Kidney autotransplantation remains a niche, although highly valuable procedure in the armamentarium of the transplant surgeon. Historically, kidney autotransplantation was first introduced by Hardy in 1963 as a salvage therapy for large ureteral injuries. Since then, it has been refined and validated in different indications including renovascular disease, ureteral strictures, and iatrogenic trauma. Despite being technically demanding, autotransplantation provides the unique advantage of kidney preservation without the need for immunosuppression. In our case, there was a ureteral defect of approximately 8 cm in the mid-ureter segment, along with a short proximal ureteral stump of only 3 cm. Our expert urologists considered a direct reconstruction or reaching with bladder lift as not feasible. A reconstruction using the Yang-Monti technique with uretero-ileo-ureterostomy could have been considered an alternative option [5]. However, this approach carries a high risk of recurrent obstruction due to mucus production and infections, as well as the need for permanent internal ureteral stenting. This is why we opted for autotransplantation. While traditionally performed via open surgery, robotic-assisted techniques have recently emerged as a minimally invasive alternative with promising outcomes [6]. Notably, Gordon et al. published the first case of totally intracorporeal robotic-assisted kidney autotransplantation, demonstrating its technical feasibility with low blood loss, minimal ischemia time, and preserved renal function at five months follow-up [7]. Robotic-assisted techniques offer distinct advantages in the context of kidney transplantation and complex autotransplantation. Compared to open procedures, they provide enhanced precision, reduced perioperative morbidity, and faster recovery times. In their review on robotic-assisted kidney transplantation, Tzvetanov et al. highlighted the benefits of smaller incisions, reduced wound complications, and superior cosmetic outcomes, which are particularly relevant for high-risk and obese patients [8]. Furthermore, the group at the University of Illinois, Chicago, demonstrated that robotic-assisted kidney transplantation enables access to transplantation for obese patients who were previously denied due to technical challenges and increased risk of surgical site infections. By reducing wound-related morbidity, this approach expanded transplant eligibility and improved equity of care [9]. This was also true for our patient, who was obese and did not experience any postoperative wound complications or surgical site infections despite the limited incisions. For both parts of the surgery, explant and implant, the same small incisions could be used, offering significant advantages in postoperative healing and recovery.

The formation of a postoperative lymphatic fistula was independent of the use of a minimally invasive technique and was more likely associated with the extensive scar tissue due to previous surgery, previous repetitively infected urinoma, and adhesions from previous mesh placement for incision hernia repair. Although resolved with conservative management, this possibly deteriorated and delayed renal recovery. This was possibly aggravated by the recurrent infections during the previous year. However, kidney function fully recovered approximately three months after the autotransplantation. In addition, the patient reported a significant improvement in quality of life without the nephrostomy, underlining the success of this surgery.

## Conclusions

Robotic-assisted kidney autotransplantation is a safe and feasible treatment option for patients with long-segment ureteral injuries for whom urological reconstruction is not possible. The minimally invasive robotic-assisted approach enables both the explantation and implantation of the kidney to be performed safely via the same small surgical access. Such procedures should only be performed at specialized centers with extensive experience in both conventional and robotic-assisted kidney transplantation.
